# The prognostic significance of postoperative neutrophil-to-lymphocyte ratio after radical prostatectomy for localized prostate cancer

**DOI:** 10.18632/oncotarget.14349

**Published:** 2016-12-29

**Authors:** Won Sik Jang, Kang Su Cho, Myung Soo Kim, Cheol Yong Yoon, Dong Hyuk Kang, Yong Jin Kang, Won Sik Jeong, Won Sik Ham, Young Deuk Choi

**Affiliations:** ^1^ Department of Urology, Urological Science Institute, Yonsei University College of Medicine, Seoul, Korea

**Keywords:** neutrophil-to-lymphocyte ratio, prostate cancer, radical prostatectomy, biochemical recurrence, survival

## Abstract

**Background:**

The pretreatment neutrophil-to-lymphocyte ratio has prognostic value after radical prostatectomy for treating localized prostate cancer. However, the use of postoperative neutrophil-to-lymphocyte ratio has not been evaluated in this population. We investigated the prognostic significance of early postoperative neutrophil-to-lymphocyte ratio after radical prostatectomy for prostate cancer.

**Methods:**

We retrospectively reviewed clinical data from 2,302 patients with localized prostate cancer who underwent radical prostatectomy at our institution between years 2000 and 2010. Only patients with pre- and postoperative complete blood counts with differential results were included. Patients who received neoadjuvant or postoperative adjuvant treatment and those without adequate medical records were excluded. Kaplan-Meier analyses were performed to analyze biochemical recurrence-free survival and overall survival rates. Univariate and multivariate Cox regression models were used for each endpoint.

**Results:**

Kaplan-Meier curves showed that high postoperative neutrophil-to-lymphocyte ratio (>3.5) was significantly associated with decreased biochemical recurrence-free survival (*p* = 0.009) and overall survival (*p* = 0.010). In the univariate and multivariate Cox regression analyses, high postoperative neutrophil-to-lymphocyte ratio was a significant predictor of biochemical recurrence (hazard ratio 1.270, *p* = 0.008) and overall survival (hazard ratio 1.437, *p* = 0.033).

**Conclusions:**

Our results demonstrate that postoperative neutrophil-to-lymphocyte ratio is an independent factor for biochemical recurrence and overall survival in patients who underwent radical prostatectomy for prostate cancer. These findings suggest that neutrophil-to-lymphocyte ratio can be a potentially valuable tool for stratifying high-risk patients and facilitating choices of postoperative therapy in patients with prostate cancer.

## INTRODUCTION

Virchow first proposed a connection between inflammation and cancer in 1876, and findings since then suggest that the host inflammatory response plays an important role in cancer development and progression [1–3]. In particular, the systemic inflammatory response, as evidenced by surrogate blood-based parameters such as C-reactive protein or circulating inflammatory blood cells, plays crucial parts in the recurrence, progression, metastasis, and survival of cancer cells in a variety of malignancies [4–6]. Several studies published in the last decade have shown that an increased neutrophil-to-lymphocyte ratio (NLR) has prognostic value in patients with localized and advanced cancers including gastrointestinal, liver, lung, breast, ovaries, and urological cancers [4, 5, 7–12].

In the case of prostate cancer (PC), many studies have shown that NLR has prognostic value in patients with metastatic castration-resistant PC receiving chemotherapy [13–15]. A few studies reported that an elevated NLR has prognostic value after radical prostatectomy (RP) for localized PC [16–18]. However, most of these focused on the prognostic value of NLR in the preoperative setting; no study has assessed the association between postoperative NLR and prognosis after RP for PC. According to some reports [12, 19, 20], the NLR after potentially curative tumor resection can better reflect a patient's systemic immune responses, which could influence cancer prognosis.

Here we tested the hypothesis that postoperative NLR can predict oncologic outcome after RP for PC. We examined clinical and pathological characteristics according to postoperative NLR during the recovery period to evaluate the influence of postoperative NLR on biochemical recurrence (BCR)-free survival and overall survival (OS) in patients following RP for PC.

## RESULTS

### Descriptive statistics

A total of 2,032 PC patients were included. The median follow-up from RP was 78 months (interquartile range [IQR] 65–95), and the median postoperative NLR was 2.3 (IQR 1.6–3.5). The median time from RP to determination of postoperative NLR was 78 days (IQR 68−84). Table 1 shows the patients’ clinical and pathological features, which are stratified into groups by postoperative NLR (high > 3.5 and low ≤ 3.5). We found significant differences in age, preoperative prostate-specific antigen (PSA) level, body mass index (BMI), and pathological stage between the groups. Patients in the high NLR group had higher neutrophil and lower lymphocyte counts. Meanwhile, we found no significant differences in hypertension, diabetes mellitus (DM), cardiovascular disease (CVD), and prior cerebral vascular accident (CVA) between the groups.

**Table 1:** Baseline patient characteristics

Variable	Total	Low group	High group	p value
	n = 2032 (%)	1579 (77.7)	453 (22.3)	
Age, years (median)	66	66	66	0.007
IQR	61–70	61–70	62–70	
PSA, ng/ml (median)	8.1	7.9	9.3	0.001
IQR	5.4–13.5	5.3–13.1	5.7–15.2	
BMI, kg/m^2^ (median)	24.1	24.2	23.7	0.004
IQR	22.3–25.7	22.5–25.7	21.9–25.4	
Hypertension				0.748
No	1118 (55.0)	872 (55.2)	246 (54.3)	
Yes	914 (45.0)	707 (44.8)	207 (45.7)	
DM				>0.999
No	1675 (82.4)	1301 (82.4)	374 (82.6)	
Yes	357 (17.6)	278 (17.6)	79 (17.4)	
CVD				0.200
No	1842 (90.6)	1424 (90.2)	418 (92.3)	
Yes	190 (9.4)	155 (9.8)	35 (7.7)	
CVA				0.421
No	1917 (94.3)	1493 (94.6)	424 (93.6)	
Yes	115 (5.7)	86 (5.4)	29 (6.4)	
Gleason score				>0.999
<7	621 (30.6)	483 (30.6)	138 (30.5)	
≥7	1411 (69.4)	1096 (69.4)	315 (69.5)	
PSM				0.632
No	1039 (51.1)	812 (51.4)	227 (50.1)	
Yes	993 (48.9)	767 (48.6)	226 (49.9)	
T stage				0.048
T2	935 (46.0)	708 (44.8)	227 (50.1)	
T3	1097 (54.0)	871 (55.2)	226 (49.9)	
LN metastasis				0.351
No	1920 (94.5)	1496 (94.7)	424 (93.6)	
Yes	112 (5.5)	83 (5.3)	29 (6.4)	
Preoperative neutrophil, ×10^3^/μl (median)	3.52	3.46	3.78	<0.001
IQR	2.83–4.44	2.79–4.33	2.99–4.73	
Preoperative lymphocyte, ×10^3^/μl (median)	1.97	2.01	1.82	<0.001
IQR	1.59–2.42	1.63–2.46	1.49–2.30	
Postoperative neutrophil, ×10^3^/μl (median)	4.01	3.62	6.92	<0.001
IQR	3.05–5.31	2.86–4.53	5.13–9.00	
Postoperative lymphocyte, ×10^3^/μl (median)	1.77	1.93	1.20	<0.001
IQR	1.36–2.25	1.56–2.37	0.96–1.52	

Abbreviations: BMI = body mass index; CI = confidence interval; CVA = cerebral vascular accident; CVD = cardiovascular disease; DM = diabetes mellitus; HR = hazard ratio; IQR = interquartile range; LN = lymph node; NLR = neutrophil to lymphocyte ratio; PSA = prostate specific antigen; PSM = positive surgical margin.

### Postoperative NLR and biochemical recurrence

**Figure 1:**
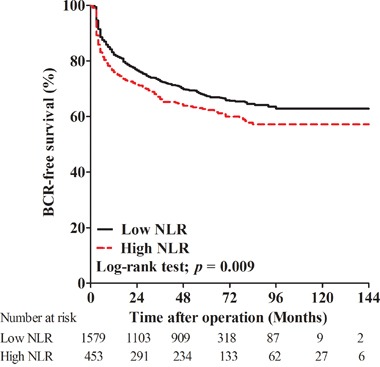
Kaplan-Meier curves for biochemical recurrence (BCR)-free survival in patients with high postoperative neutrophil to lymphocyte ratio (NLR) and low NLR

In all, 665 (32.7%) men had experienced BCR. There were 495 (31.3%) and 170 (37.5%) patients with BCR in low and high NLR groups, respectively. The 5-year BCR-free survival rates were 67.4% and 62.4%, while the 10-year BCR-free survival rates were 62.9% and 57.2% in low and high NLR groups, respectively. Kaplan-Meier curves showed that the BCR-free survival rate for men with high NLR was worse compared to men with low NLR (log-rank test; p = 0.009, Figure 1).

In both univariate and multivariate Cox regression analyses, patient's age (hazard ratio [HR] 1.012, p = 0.040), preoperative PSA (HR 1.004, *p* < 0.001), Gleason score (HR 3.069, p <0.001), positive surgical margin (PSM; HR 1.992, *p* < 0.001), T-stage (HR 1.469, *p* < 0.001), lymph node (LN) metastasis (HR 1.535, p = 0.002), and NLR (HR 1.270, *p* = 0.008) were all independent prognostic factors for BCR (Table 2).

**Table 2:** Univariate and multivariate analyses of factors associated with biochemical recurrence

Variable	Univariate		Multivariate	
	HR (95% CI)	*p* value	HR (95% CI)	*p* value
Age	1.024 (1.012–1.035)	<0.001	1.012 (1.001–1.024)	0.040
PSA	1.005 (1.004–1.006)	<0.001	1.004 (1.003–1.005)	<0.001
BMI	0.981 (0.951–1.012)	0.236		
Hypertension				
No	1 (Ref)			
Yes	1.055 (0.906–1.229)	0.489		
DM				
No	1 (Ref)			
Yes	1.180 (0.974–1.431)	0.091		
CVD				
No	1 (Ref)			
Yes	1.221 (0.956–1.560)	0.109		
CVA				
No	1 (Ref)			
Yes	1.077 (0.776–1.495)	0.656		
Gleason score				
<7	1 (Ref)		1 (Ref)	
≥7	4.240 (3.342–5.379)	<0.001	3.069 (2.400–3.924)	<0.001
PSM				
No	1 (Ref)		1 (Ref)	
Yes	2.820 (2.392–3.324)	<0.001	1.992 (1.669–2.376)	<0.001
T stage				
T2	1 (Ref)		1 (Ref)	
T3	2.546 (2.152–3.013)	<0.001	1.469 (1.222–1.765)	<0.001
LN metastasis				
No	1 (Ref)		1 (Ref)	
Yes	1.910 (1.449–2.518)	<0.001	1.535 (1.163–2.027)	0.002
Delta NLR				
Decrease	1 (Ref)			
Increase	1.162 (0.986–1.369)	0.074		
Postoperative NLR				
Low	1 (Ref)		1 (Ref)	
High	1.259 (1.057–1.499)	0.010	1.270 (1.066–1.514)	0.008

Abbreviations: BMI = body mass index; CI = confidence interval; CVA = cerebral vascular accident; CVD = cardiovascular disease; DM = diabetes mellitus; HR = hazard ratio; LN = lymph node; NLR = neutrophil to lymphocyte ratio; PSA = prostate specific antigen; PSM = positive surgical margin.

### Postoperative NLR and overall survival

**Figure 2:**
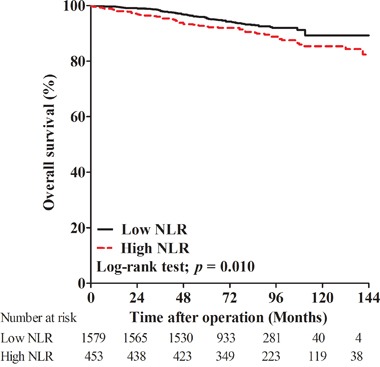
Kaplan-Meier curves for overall survival in patients with high postoperative neutrophil to lymphocyte ratio (NLR) and low NLR

In the current study, 160 (7.9%) men died during follow-up due to all possible causes. Of these deaths, 103 (6.5%) patients were from the low NLR group and 57 (12.6%) were from the high NLR group. The 5-year OS rates of the low and high NLR groups were 95.4% and 92.5%, while the 10-year OS rates were 89.3% and 85.4%, respectively. Kaplan-Meier curves showed that OS for men with high NLR was worse than those with low NLR (log-rank test; *p* = 0.010, Figure 2).

In our univariate and multivariate Cox regression analyses, patient's age (HR 1.079, *p* < 0.001), DM (HR 1.476, *p* = 0.033), Gleason score (HR 1.643, *p* = 0.014), LN metastasis (HR 2.336, *p* < 0.001), and NLR (HR 1.437, *p* = 0.033) were all independent prognostic factors for OS (Table 3).

**Table 3:** Univariate and multivariate analyses of factors associated with overall survival

Variable	Univariate		Multivariate	
	HR (95% CI)	*p* value	HR (95% CI)	*p* value
Age	1.085 (1.057–1.113)	<0.001	1.079 (1.051–1.108)	<0.001
PSA	1.003 (1.000–1.006)	0.077		
BMI	0.960 (0.897–1.029)	0.249		
Hypertension				
No	1 (Ref)			
Yes	1.204 (0.883–1.642)	0.240		
DM				
No	1 (Ref)			
Yes	1.606 (1.126–2.290)	0.009	1.476 (1.032-2.110)	0.033
CVD				
No	1 (Ref)			
Yes	1.553 (0.981–2.458)	0.060		
CVA				
No	1 (Ref)			
Yes	1.729 (0.999–2.992)	0.050		
Gleason score				
<7	1 (Ref)		1 (Ref)	
≥7	1.949 (1.316–2.885)	0.001	1.643 (1.106–2.440)	0.014
PSM				
No	1 (Ref)		1 (Ref)	
Yes	1.478 (1.080–2.024)	0.015	1.214 (0.875–1.684)	0.245
T stage				
T2	1 (Ref)		1 (Ref)	
T3	1.413 (1.028–1.942)	0.033	1.019 (0.706–1.470)	0.922
LN metastasis				
No	1 (Ref)		1 (Ref)	
Yes	2.498 (1.564–3.992)	<0.001	2.336 (1.457–3.744)	<0.001
Delta NLR				
Decrease	1 (Ref)			
Increase	0.913 (0.650–1.283)	0.600		
Postoperative NLR				
Low	1 (Ref)		1 (Ref)	
High	1.548 (1.109–2.160)	0.010	1.437 (1.029–2.007)	0.033

Abbreviations: BMI = body mass index; CI = confidence interval; CVA = cerebral vascular accident; CVD = cardiovascular disease; DM = diabetes mellitus; HR = hazard ratio; LN = lymph node; NLR = neutrophil to lymphocyte ratio; PSA = prostate specific antigen; PSM = positive surgical margin.

## DISCUSSION

Several studies suggested that a systemic inflammatory response is associated with a poor oncologic outcome in multiple cancers [4–6]. NLR is a widely used systemic inflammatory marker because of its low cost and wide availability in clinical practice. Although the exact mechanism underlying the poor prognostic impact of an elevated NLR remains unclear, this association may relate to increased neutrophil-dependent inflammation and a decreased lymphocyte-mediated tumor response [21]. Malignant tumor cells secrete a variety of pro-inflammatory cytokines and negative modulators of anti-tumor immunity that may cause relative neutrophilia and lymphocytopenia. Neutrophils contribute to enhanced angiogenesis and tumor cell intravasation [22]. In addition, circulating neutrophils produce inflammatory mediators such as tumor necrosis factor and interleukin, which promote tumor cell proliferation and angiogenesis [23]. Lymphocytes are involved in cytotoxic cell death and cytokine production, which inhibits tumor cell proliferation and metastasis [24]. The presence of lymphocytes in a tumor is associated with better responses to cytotoxic treatment and a more favorable prognosis among cancer patients [25].

NLR was previously shown to predict metastatic castration-resistant PC survival [13–15]. Several recent studies suggested that elevated NLR can be an independent prognostic factor for oncological outcomes in localized PC [16–18, 22]. Although there were a few studies dealing with relationship between NLR and BCR after RP, the reported cut-off values were inconsistent [16–18, 26, 27]. Minardi et al. reported that patients with high NLRs had a greater incidence of BCR [18]. Moreover, Lee and colleagues retrospectively analyzed 1367 patients and found that a high NLR was significantly related to unfavorable clinicopathological outcomes and shorter BCR-free survival [17]. Others reported that high NLR was associated with higher BCR risk. Our previous study found that a high preoperative NLR was associated with overall and cancer-specific mortality but not with BCR [16]. Similarly, Bahig et al. demonstrated that NLR was not associated with BCR after RP for localized PC [26]. Kwon et al. determined that BCR after RP was not significantly different between the high and low NLR groups [27].

Given the inconsistent BCR results across studies, we decided to focus on the prognostic value of NLR in the postoperative setting. Our results show that elevated postoperative NLR is a useful independent prognostic factor for BCR and OS in patients with localized PC. Similarly, Kang et al. reported that postoperative NLR was a significant predictive factor for survival outcomes in patients with urothelial carcinoma of the bladder undergoing radical cystectomy [12]. Li et al. suggested that the postoperative NLR combined with the platelet-to-lymphocyte ratio predicted outcomes of hepatitis B virus-related hepatocellular carcinoma patients after liver resection [19]. Shibutani et al. reported that the postoperative NLR is an independent prognostic factor in patients with colorectal cancer who underwent potentially curative surgery [28].

NLR reflects the balance between innate (neutrophil-mediated) and adaptive (lymphocyte-mediated) immune responses [11]. Thus, high and low NLR values after RP suggest pro- and anti-tumor inflammatory responses, respectively. Postoperative NLR could therefore reflect residual host immune activity after RP. In addition, surgical stress is a systemic response to surgical injury and is characterized by sympathetic nervous system activation, endocrine responses, and immunological and hematological changes [29]. Such stress could shift the host systemic inflammatory state in favor of tumor recurrence and growth after RP [19]. In this regard, postoperative NLR can reflect how a patient's systemic immune activity has changed after surgery. Both the pre- and postoperative NLR values should be considered when predicting the prognosis of patients with PC who underwent RP.

There are several strengths to our study. First, to our best knowledge, this is the first investigation of the prognostic significance of postoperative NLR in patients with PC who underwent RP. Second, our cohort included a large number of patients with sufficiently long-term follow-up durations, which allowed us to show that a high postoperative NLR was significantly associated with both short- and also long-term survival. Lastly, our postoperative follow-up protocol was homogeneous. No patient received adjuvant androgen deprivation therapy or radiotherapy until BCR, which allowed us to observe the natural history of BCR after RP.

Our analysis also has several limitations. First, this was a retrospective review of data from patients treated at a single institution, so our results may not be generalizable. Second, after cancer recurrence, patients in our study were treated based on their clinician's practices. Third, other systemic inflammation markers such as C-reactive protein and albumin were not routinely available and could not be included in our analysis. Further prospective, multicenter studies are needed to confirm our results.

In conclusion, our study demonstrates that postoperative NLR is an independent prognostic factor for BCR and OS in patients who underwent RP for PC. As our findings suggest, we believe NLR can be a valuable tool for not only stratifying high-risk patients, but also for facilitating the decision-making of postoperative therapy for patients with prostate cancer.

## MATERIALS AND METHODS

### Patients

After obtaining approval from the Institutional Review Board of the Severance Hospital, we retrospectively reviewed medical records of 2,302 patients, with clinically localized or locally advanced PC, who underwent RP at our institution between January 2000 and December 2010. Among these patients, we only considered those who had perioperative leukocyte parameters from peripheral blood including neutrophil, lymphocyte, and total leukocyte counts. We excluded patients with a history of double primary cancers, autoimmune or systemic inflammatory diseases that may influence NLR values, as well as those with suspected bacterial or viral infection by laboratory results. We also excluded patients under neoadjuvant or adjuvant therapy, and those with incomplete medical records.

### Patient characteristics

Clinical characteristics including age, PSA at the time of biopsy, BMI, pathological Gleason score, PSM, pathological T stage, and LN metastasis were obtained through a review of medical records at our institution. TNM stage was determined according to the 7^th^ edition of the American Joint Committee on Cancer TNM staging system [30]. Pathological analysis of RP specimens was performed by a single experienced uropathologist. We included the data on metabolic comorbidities (hypertension, DM, CVD and a prior CVA) associated with systemic inflammation, which are important predictors of long-term survival outcomes in patients with prostate cancer. For perioperative leukocyte parameters counts, preoperative values were obtained from routine laboratory tests within one month before RP, while postoperative counts were taken during the recovery period of two to three months after RP. Delta NLR was defined as postoperative NLR minus preoperative NLR [19].

### Follow-up

Postoperative PSA follow-up was performed monthly for the first 6 months, every 3 months for the second year, and semiannually thereafter. BCR was defined as any two consecutive increases in serum PSA ≥0.2 ng/ml following RP [31]. BCR-free survival was defined as the time from RP to BCR. Mortality data were obtained from medical records in our institution's Cancer Registry Center database [32]. The follow-up period was calculated from the time of RP to the date of the last known contact with the patient or the date of death. OS was defined as the time from RP to the date of death by any cause.

### Statistical analysis

The NLR was calculated by dividing the neutrophil count by the lymphocyte count. To determine the optimal cut-off value of NLR, we performed survival analysis with the Kaplan–Meier method with a log-rank test based on the NLR quartiles (1st quartile, NLR ≤1.6; 2nd quartile, 1.6 < NLR ≤ 2.3; 3rd quartile, 2.3 < NLR ≤ 3.5; 4th quartile, NLR >3.5). There was a significant difference in BCR-free survival for the top 25^th^ percentile (NLR cut-off of 3.5) when the cohort was dichotomized by quartiles. We compared clinical and pathological characteristics between groups using Mann–Whitney U tests for continuous data and χ^2^ tests for dichotomous variables. We used the Kaplan-Meier method with log-rank tests to estimate and compare oncologic outcomes according to NLR group. Cox proportional hazard models were used to investigate associations between variables and the risks of oncologic outcomes. Significant variables from univariate analysis were included in the multivariate analysis. All analyses were conducted using SPSS 18.0 software (SPSS, Inc., Chicago, IL, USA). Comparisons with *p* < 0.05 were considered statistically significant.
